# Supplementary material for: Pathophysiological Significance of Neutrophilic Transfer RNA-Derived Small RNAs in Asymptomatic Moyamoya Disease
Source: Cells. 2021 May 1;10(5):1086. doi: 10.3390/cells10051086 (PMC8147334; doi:10.3390/cells10051086)

2D Structure | **Local AU** | **Position**

```
144                                   8mer-1a      178
 5'-ctccATCCTTAACTGTCCAGTCCAGTGATGTGGGa-3'  utr
        |||| ||||:|  | || ||     |||||||
 3'-uccuUAGG-AUUGGC-GAUCUGG----UACACCCu-5'  tRF
                 16 151413        765432
              3' Pairing            Seed
```

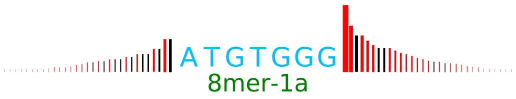

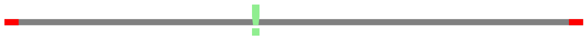

Supplement: Supplementary file 1 [file cells-10-01086-s001.zip › cells-1149161 3rd supplementary materials/Data Supplements/Data Supplement V.zip!/Data supplement 6-mRNAs targets of tsRNAs/tsRNA-Glu-TTC-010/Diagrams/tRF-Glu-TTC-010_vs_ENST00000371418(LGI1).pdf]
